# Aortic root repair in a patient with acquired hemophilia A: case report

**DOI:** 10.1186/s40792-021-01256-x

**Published:** 2021-08-04

**Authors:** Emre Gok, Mehmet H. Akay, Ismael Salas de Armas, Kimberly Klein, Hlaing Tint, Paul M. Allison, Alice J. Chen, Bindu Akkanti, Biswajit Kar, Igor D. Gregoric

**Affiliations:** 1 Center for Advanced Heart Failure, University of Texas Health Science Center at Houston, 6400 Fannin, Suite 2350, Houston, TX 77030 USA; 2 Gulf Coast Pathology Associates, Houston, TX 77339 USA

**Keywords:** Case report, Aortic root repair, Hemophilia, Factor VIII

## Abstract

**Background:**

Patients with acquired hemophilia A (AHA) who require open heart surgery have a life-threatening risk of hemorrhage. Limited data exist to guide perioperative management of these patients.

**Case presentation:**

A 53-year-old female with rheumatoid arthritis, concomitant aortic valve endocarditis, and severe aortic regurgitation presented to our hospital. Bleeding and abnormal coagulation tests were noted during the initial workup, and she was diagnosed with AHA. The perioperative management plan included the use of pharmaceuticals, porcine recombinant factor VIII, and blood products. Extensive preoperative coagulation data were obtained, and factor VIII levels were continuously monitored to mitigate bleeding complications. The aortic valve replacement and root repair were uneventful.

**Conclusion:**

Cardiac surgery in patients with AHA is possible as long as complex perioperative hemostatic and hematology management is used.

## Background

Acquired hemophilia A (AHA) is a rare autoimmune disease where autoantibodies decrease the activity and plasma half-life of coagulation factor VIII (FVIII), resulting in bleeding [1]. Rheumatoid arthritis (RA), administration of tumor necrosis factor (TNF) alpha inhibitors, and underlying malignancies have all been associated with FVIII autoantibody formation [2]. As these associated risk factors are all more frequently observed in older adults, the same population that is at greater risk for cardiovascular disease, it is critical that guidance be provided for perioperative management of patients with AHA. Limited data are available to guide perioperative management of patients with AHA. Herein, we describe the diagnosis of AHA alongside the perioperative management of cardiac surgery.

## Case presentation

A 53-year-old female with RA presented with edema in the upper extremities and neck, cough, and orthopnea. She was receiving anti-TNF therapy (over one year) and infliximab (over 1 month).

**Fig. 1:**
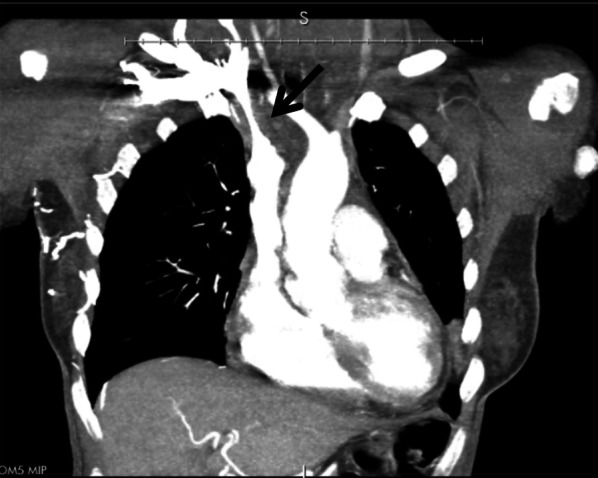
A computed tomography scan of the chest identified significant mediastinal lymphadenopathy (arrow) that compressed the airway and the superior vena cava

The patient’s initial clinical picture was consistent with superior vena cava compression syndrome. Imaging revealed significant mediastinal lymphadenopathy that was compressing the airway and the superior vena cava (Fig. 1). An echocardiogram showed aortic valve vegetation and severe aortic regurgitation (AR). Thus, her acute decompensated congestive heart failure symptoms were due to severe aortic insufficiency. The patient denied a history of fever and blood cultures were negative. The multidisciplinary team agreed that the most likely cause was non-bacterial thrombotic endocarditis. The patient was treated with empiric vancomycin and ceftriaxone, and steroids were initiated. A supraclavicular lymph node biopsy was negative for malignancy; however, ecchymosis developed throughout her neck after the procedure. Intravenous solumedrol was started to reduce the swelling.

### Diagnosis of AHA

**Fig. 2:**
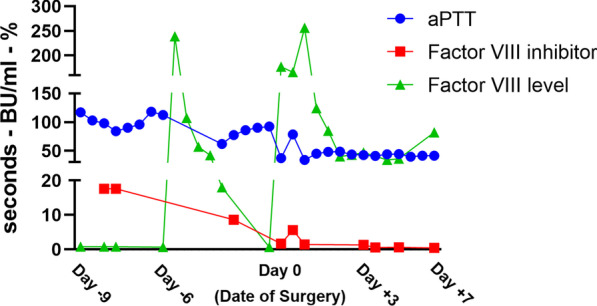
Laboratory data demonstrating the therapeutic response of porcine recombinant Factor VIII

Coagulation testing noted a prolonged activated partial thromboplastin time (aPTT). An anti-Xa assay was performed to confirm the absence of any systemic anticoagulation. A subsequent mixing study with normal plasma failed to improve the PTT (90.5 s after one hour) indicating the presence of a factor inhibitor. Lupus testing was negative; however, the analysis of coagulation factors revealed an absence of FVIII and the presence of a FVIII inhibitor (quantified at 17.60 Bethesda units). These results, along with normal von Willebrand factor antigen and activity, resulted in the diagnosis of AHA (Fig. 2).

### Perioperative management

A trial dose of porcine recombinant FVIII (rFVIII) was given and initiated a robust response (peak levels of FVIII = 239). The ecchymosis in her neck improved; however, her cardiopulmonary condition deteriorated. Due to severe AR and an aortic root abscess, an urgent aortic valve repair (AVR) was deemed necessary. The preoperative Society of Thoracic Surgeon’s risk score was 18.39%.

An additional dose (3728 units) of the rFVIII was given 1 h prior to surgery. After anesthesia, 1 g of tranexamic acid (TXA) was given intravenously; 2.5 g of TXA was given during the course of the surgery. The cardiopulmonary bypass pump (CBP) was primed with 1.048 L of fresh frozen plasma (FFP), and 12,000 IU of systemic heparin was given. The activated clotting time (ACT) was 582 s. Following cannulation, CBP was initiated. Retrograde autologous priming was performed. The procedure was performed under aortic crossclamp, cardioplegia, and mild hypothermia. The AVR was performed with a bioprosthetic valve and root reconstruction using a pericardial patch.

Upon warming, the hematocrit level was 22 mg/dl even after 1250 mL of hemoconcentration; therefore, we added 1 unit of packed red blood cells (pRBCs). After termination of CPB, 130 mg of protamine normalized the ACT (95 s). The patient received an additional 1 unit of pRBCs, 1 unit of FFP, and 1 unit of platelets. Moreover, she received an additional dose of rFVIII (1900 units). The timing and dosage of the post-CPB rFVIII was based on preoperative coagulation data. According to serial FVIII levels (Fig. 2), the patient cleared 50% of the rFVIII in approximately 4–6 h. This preoperative information was valuable as it provided guidance on how to achieve target FVIII levels (100 IU/dL) and hemostasis during the procedure. Estimated blood loss was 1300 cc, and she received 450 mL of salvage blood. The procedure was uneventful, and she was transferred to the cardiac unit.

The patient experienced no significant bleeding events after the procedure and was extubated on postoperative day (POD) 1. A bolus of FVIII was given every 6 h during the first postoperative 18 h to ensure that the FVIII level remained about 50%. The patient received an additional bolus of FVIII at postoperative hour 30. Rituximab was added to prednisolone to eliminate the FVIII inhibitor. On POD 3, FVIII activity was 85 indicating a favorable response, and all chest tubes were removed. Her postoperative course was uneventful. A continuous decline of the FVIII inhibitor and normalization of the PTT was observed. She was discharged at POD 10.

## Conclusion

**Fig. 3:**
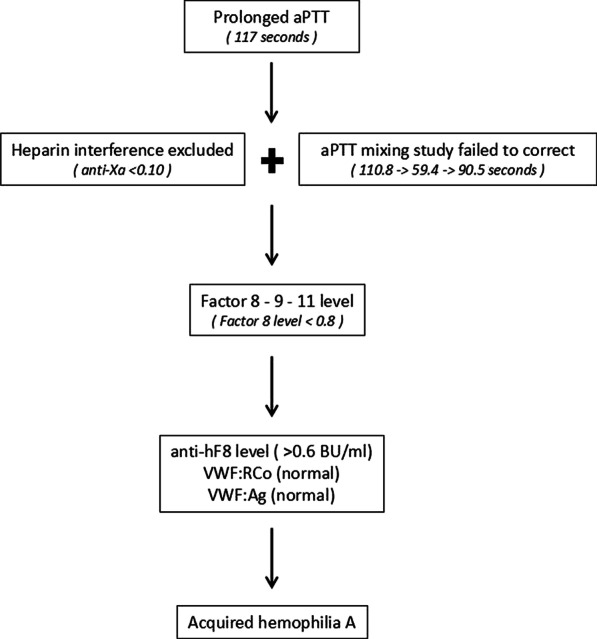
Diagnostic steps for acquired hemophilia A. *Ag* antigen; *RCo* ristocetin, *VWF* von Willebrand factor

The incidence of AHA is extremely low (1.48 cases per million), and FVIII levels and inhibitor titers in these patients fluctuate; thus, assessments at presentation are often not useful for predicting the severity of bleeding [2]. Cardiac surgery is often performed under CPB for which systemic anticoagulation with heparin is necessary; coagulopathy after CPB is complex and affects the perioperative management of AHA patients as they already suffer bleeding manifestations. It is paramount to diagnose AHA (Fig. 3) and carry out diligent perioperative management (bleeding control, reversal of anticoagulation after CPB, and eradication of the inhibitor factor) [3] in these cases (Table 1). This case report summarizes key elements of optimal management of this challenging clinical scenario.

**Table 1:** Perioperative management for cardiac surgery in patients with acquired hemophilia A [4, 5]

Preoperative	Check baseline FVIII and FVIII inhibitor levels
	Check baseline standard clotting tests
	Establish two reliable venous access points—one for sampling and the other for administration of FVIII
	Infuse a trial of porcine rFVIII and evaluate therapeutic response for dose adjustment
	Give bolus of the calculated porcine rFVIII to achieve target FVIII level of 100 IU/dL (administered 2–3 h before surgery)
Intra-operative	Check FVIII level prior to first incision (target > 100 IU/dL)
	Repeat FVIII level checks before heparinization—give bolus of rFVIII if level is < 100 IU/dL
	Repeat FVIII level checks every 30 min during CPB—patient will need chromogenic FVIII/heparin neutralization in laboratory Check FVIII level immediately after CPB and after protamine infusion—give bolus if level is < 100 IU/dL)
Postoperative	Repeat FVIII level checks at 4- to 6-h intervals during early postoperative period
	Check FVIII levels 2 times/day during the first 3 postoperative days and less frequently thereafter Give bolus of rFVIII if required

*CPB* cardiopulmonary bypass, *FVIII* Factor VIII, *rFVIII* recombinant Factor VIII

Failure to obtain stable clotting in AHA patients may lead to life-threatening hemorrhage or thrombotic complications. Currently, there are two main hemostatic options: bypassing agents (activated prothrombin complex concentrates and recombinant activated factor VIIa) and immunoadsorption with porcine rFVIII infusions [6]. We recommend the latter for patients undergoing cardiac surgery as its hemostatic efficacy was evident in our case as well as others [7–9]. We believe that porcine rFVIII is more effective than human-based versions for acquired hemophilia A because the porcine factor VIII’s protein sequence differs from human factor VIII and may be less inactivated by factor VIII inhibitors. In a prospective study by Kruse-Jarres et al., 28 patients with an acquired factor VIII inhibitor and severe bleeding were treated with OBI-1 (an antihemophilic factor VIII that uses the porcine sequence) [10]. This product controlled bleeding in 24 individuals (86%). Efficacy was greater in those who received OBI-1 as a primary therapy versus those who received another hemostatic agent first (94% versus 73%). Patient comorbidities included a variety of underlying conditions such as malignancy or rheumatologic disease; no underlying etiology for acquired factor VIII inhibitor was identified in 16 (57%) patients [10].

Importantly, FVIII levels can be monitored so the response to rFVIII treatment can be tracked, and thrombotic complications might be prevented. Furthermore, the hemostatic management of patients with AHA via bypassing agents can be challenging during CPB, as they have short plasma half‐lives; thus, multiple infusions are required for anticoagulation reversal [4]. In addition, bypassing agents carry a risk of thrombotic events [11], and there is not a test to monitor under- or overdosing [6].

Autoantibody removal by plasmapheresis, immunoadsorption with staphylococcal protein A, or polyclonal sheep antibodies against human immunoglobulins might be considered as alternative strategies. However, there is limited data about their efficacy, and they might be restricted due to the hemodynamic instability, infection, or anticoagulation [12].

In conclusion, when cardiac surgery is unavoidable in patients with AHA, FVIII levels must be increased prior to the procedure. Cardiac surgery is possible as long as complex perioperative hemostatic management with pharmaceuticals, FVIII agents, and blood products are utilized.

## Data Availability

The patient’s electronic medical record is the source material for this study. A de-identified dataset is available upon request.

## Abbreviations

ACT: Activated clotting time
Ag: Antigen
AHA: Acquired hemophilia A
aPTT: Activated partial thromboplastin time
AR: Aortic regurgitation
AVR: Aortic valve repair
CBP: Cardiopulmonary bypass pump
FFP: Fresh frozen plasma
FVIII: Factor VIII
POD: Postoperative day
pRBCs: Packed red blood cells
RA: Rheumatoid arthritis
RCo: Ristocetin
rFVIII: Recombinant FVIII
TNF: Tumor necrosis factor
TXA: Tranexamic acid
VWF: Von Willebrand factor
